# Effect of By-Products from Pistachio Skin on Gastrointestinal Microbiota of Healthy Lambs as Sustainable Feeding Ingredient

**DOI:** 10.3390/microorganisms14020358

**Published:** 2026-02-03

**Authors:** Georgiana Bosco, Amanda Vaccalluzzo, Nunziatina Russo, Alessandra Pino, Cinzia Caggia, Cinzia Lucia Randazzo

**Affiliations:** 1 Department of Agriculture, Food and Environment, University of Catania, 95123 Catania, Italy; georgiana.bosco@phd.unict.it (G.B.); nunziatinarusso83@gmail.com (N.R.); cinzia.caggia@unict.it (C.C.); cinzia.randazzo@unict.it (C.L.R.); 2 ProBioEtna srl, Spin off of the University of Catania, 95123 Catania, Italy; amanda.vaccalluzzo@unict.it

**Keywords:** pistachio by-products, sustainable feeding, stool samples, ruminal samples, metagenomics

## Abstract

Pistachio skin is a by-product that is considered a promising novel feed ingredient for ruminants; however, its role in shaping the lamb gastrointestinal tract microbiota is poorly studied. The present study aimed to investigate, through a metagenomics approach, the effects of integrating pistachio skin into the diet on the faecal and ruminal microbiota of healthy lambs. Faecal samples, collected at the beginning (d0) and 58 days after the start of the dietary treatment (d58), and ruminal samples, collected after slaughter, were subjected to Illumina MiSeq analysis of the 16S rRNA gene. The results revealed that, although temporal variations were observed, the supplementation of pistachio skin did not markedly affect the overall faecal microbiota structure. Conversely, specific rumen taxa were selectively modulated by the experimental diet. In conclusion, the use of pistachio skin as a feed ingredient can be considered a suitable and sustainable dietary strategy that modulates specific rumen microbial groups, thereby preserving the stability of the gut microbiota in lambs.

## 1. Introduction

The ruminant gastrointestinal tract hosts a highly diverse microbial community that plays essential roles in digesting dietary substrates and modulating host immune function [1,2,3]. In particular, the faecal microbiota reflects the final stages of digestion and is more influenced by hindgut fermentation [4], while the rumen microbiota mainly focuses on fermenting complex nutrients, producing volatile fatty acids that are crucial for energy [5]. Several factors, including host age, diet, genetics, and environmental interactions, affect the dynamics and composition of the gastrointestinal microbiota. Notably, diet is one of the main drivers of both faecal and ruminal microbial communities, influencing fermentation pathways, nutrient availability, immune responses, and overall animal performance. Recently, incorporating by-products from agro-industrial processes into animal diets has emerged as a promising way to promote sustainable livestock systems. This strategy could lower disposal costs and enhance animal welfare [6,7]. Agro-industrial by-products, often inedible to humans, contain dietary fibre and bioactive compounds that can improve intestinal health and exert antimicrobial properties, thereby reducing the risk of disease [6,8]. Including these by-products in ruminant diets may also support environmental sustainability by diverting large amounts of agricultural waste from landfills, reducing pollution, and advancing circular economy principles [9,10]. In this context, by-products from *Pistacia vera* L. (pistachio) serve as a natural source of bioactive molecules, such as polyphenols and tannins. Although excessive intake of these compounds can cause them to act as antinutritional factors, with adverse effects on growth performance, research has investigated the inclusion of a mixture of hulls, twigs, leaves, and parts of the shell and kernel in the diets of ruminants, growing calves, dairy goats, and lambs [11,12,13,14,15]. Among pistachio by-products, the skin is considered a promising new feed ingredient for ruminants due to its high content of dietary fibre, polyphenols, flavonoids, anthocyanins, and condensed tannins, which are key in modulating both the intestinal and ruminal microbiota and exerting systemic effects [16,17]. To date, there is limited information about using pistachio skin as animal feed [18,19,20]. Recent research by Musati and colleagues [20], using *in vitro* ruminal fermentation and biohydrogenation (BH) studies with increasing amounts of pistachio skin (70, 140, and 210 g/kg DM), found no effects on fermentation parameters such as gas and methane production, ammonia levels, or volatile fatty acid synthesis. They also showed that adding pistachio skin increased the levels of desirable monounsaturated and polyunsaturated fatty acids, including vaccenic acid, although a concomitant increase in undesirable fatty acids, such as C18:1 t10 and C18:2 t10 c12, was registered [20]. Furthermore, the effects of including 120 g/kg DM of pistachio skin in the lamb diet on growth performance and meat quality parameters were evaluated [21]. In detail, twenty-four Valle del Belice × Comisana male lambs were divided into two experimental treatments (*n* = 12) and fed *ad libitum* for 60 days. The control diet, mainly based on maize–barley, and the experimental diet, in which maize and soybean meal were partially replaced with 12% of pistachio skin, were detailed in Musati and co-workers’ work [21]. Data on the experimental diet showed higher concentrations of polyphenols and tannins with respect to the control one; these compounds are known to exhibit antimicrobial activity, suggesting a possible influence on the gastrointestinal microbiota composition. Accordingly, the present study is based on the hypothesis that dietary supplementation may influence the composition and dynamics of the gastrointestinal microbiota of lambs. Specifically, the aim of the present explorative work was to provide the preliminary characterisation of the faecal and ruminal microbial communities and to assess, within each gastrointestinal compartment, differences associated with control or experimental diets. Additionally, within each group, changes over time in the faecal microbiota composition were exploratorily evaluated. For this purpose, faecal and rumen samples, collected during a previously published *in vivo* feeding trial conducted by Musati and co-workers [21], were investigated using a metagenomics approach.

## 2. Materials and Methods

### 2.1. Faecal and Rumen Sample Collection, DNA Isolation, and Sequencing

Faecal samples were directly recovered from the rectal ampoules of lambs allocated to both control (C) and treated (T) groups, before (d0) and 58 days after the start of the feeding treatment (d58). Samples were collected at the pilot farm of the University of Catania (37°24′35.3″ N 15°03′34.9″ E) and transferred, under refrigerated conditions, to the Laboratory of Microbiology. Rumen samples were collected from each lamb immediately after slaughter. In detail, the rumen content was squeezed through four layers of cheesecloth to separate the liquid fraction; transferred into sterile plastic containers; and transported, under refrigerated conditions, to the Laboratory of Microbiology. Both faecal and rumen samples were subjected to total genomic DNA extraction using the commercial QIAamp^®^ Fast DNA Stool Mini Kit (Qiagen, Hilden, Germany), following the manufacturer’s instructions, with a slight modification consisting of a repeated bead beating (RBB) pre-treatment step [22]. The DNA concentration was evaluated using the Qubit 4.0 fluorometer (Invitrogen, Carlsbad, CA, USA). For the in-depth study of the microbiota composition, the V3–V4 region of the 16S rRNA gene was amplified and sequenced on an Illumina MiSeq platform at the facilities of Synbiotec (Camerino, Italy). The raw sequencing data for faecal and rumen samples have been deposited in the NCBI Sequence Read Archive (SRA) under the accession codes PRJNA1115961 and PRJNA1368722, respectively.

### 2.2. Bioinformatics Analysis

Raw reads were quality-checked using the FastQC [23] application; then, the bioinformatics analysis was conducted using the QIIME2 v2020.8.0 suite [24]. The reads were processed through several steps, including dereplication, removing chimeric constructs, paired-end joining, and sequence clustering, performed using the DADA2 module integrated within QIIME2 [25]. Taxonomic classification was performed using a Naïve Bayes classifier implemented in QIIME 2, trained on Greengenes2 full-length 16S rRNA gene reference sequences (release 2022.10; MD5: 98d34227fe67b34f62b464466cca4ffa), which succeeded the legacy Greengenes 13_8 reference. Greengenes2 provides an updated, genome-informed taxonomy for bacterial and archaeal 16S rRNA gene profiling. The analysis was restricted to bacterial sequences, in accordance with the experimental design [26]. The impact of the sequencing coverage depth on the microbial diversity within samples was assessed by analysing rarefaction curves, using the “diversity” module in QIIME. Subsequently, alpha diversity metrics, including the Shannon index and the 1-Simpson, were computed. Beta diversity was calculated using the Bray-Curtis dissimilarity. Ordinations were visualised through principal coordinate analysis (PCoA). Group differences in alpha diversity were evaluated using Wilcoxon rank-sum tests, while differences in beta diversity were assessed via PERMANOVA (999 permutations) using the R package vegan (v2.6-4) [27]. To compare taxonomic compositions, QIIME2 feature tables were collapsed at different taxonomic levels (phylum, family, genus). Taxa with incomplete genus-level annotations were removed before differential testing. For genus-level differential abundance between or within groups, MaAsLin2 (v1.12.0) using total sum scaling (TSS) normalisation and log transformation [28] was applied. Only genera with non-zero values across samples and meeting minimum prevalence thresholds were retained. For differential abundance analyses, performed with MaAsLin2, *p*-values were corrected for multiple testing using the Benjamini–Hochberg false discovery rate (FDR) procedure. Associations with adjusted *p*-values ≤ 0.1 were considered statistically significant. Given the limited sample size of the present study (24 animals, 12 per group), a false discovery rate (FDR) threshold of ≤0.1 was selected as a balanced criterion for exploratory analyses, allowing the control of false positives and avoiding an excess of false negatives that can arise from more conservative cutoffs (0.05) under low power.

## 3. Results

### 3.1. Faecal Microbiota

#### 3.1.1. Taxonomy Classification

A total of 3.044.572 high-quality sequences were obtained by QIIME2. In detail, the sequencing of faecal samples collected from the C group at d0 and d58 produced 791,760 and 780,202 reads, respectively. For faecal samples collected from the T group, 654,378 reads were obtained at d0 and 818,232 at d58. The sample metadata, denoising statistics, and relative abundances at each taxonomic level (phylum, family, and genus) are reported in Supplementary Data S1.

**Figure 1:**
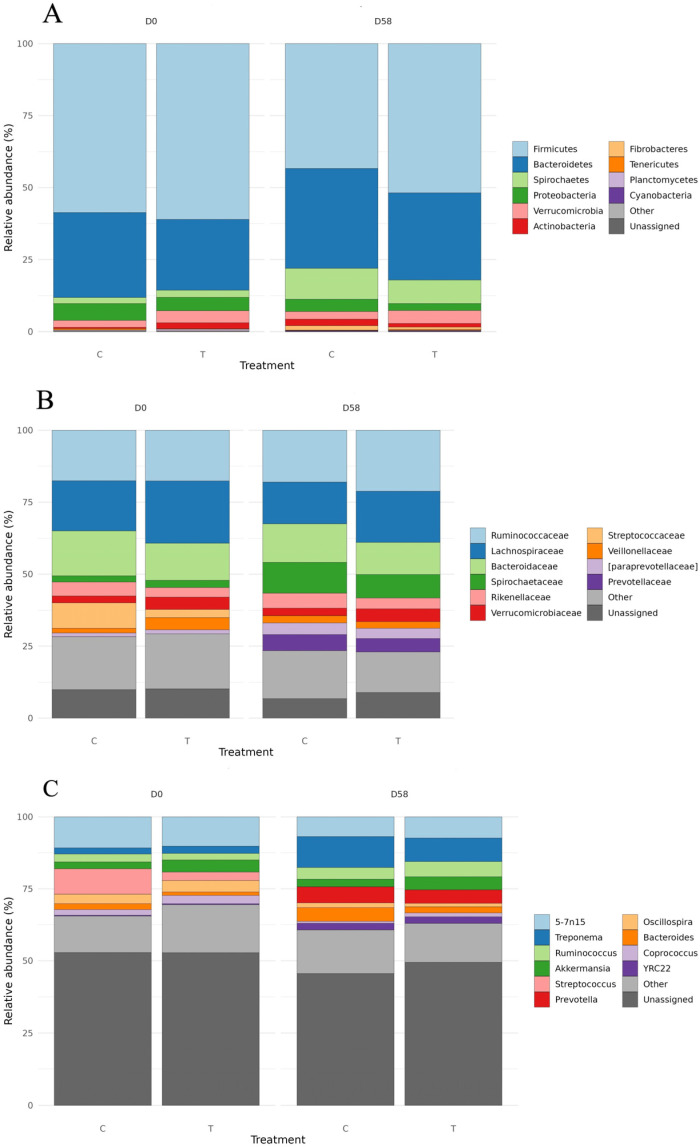
Top 10 phyla (**A**), families (**B**), and genera (**C**) detected in faecal samples collected from both control (C) and treated (T) groups at the beginning (d0) and at the end of the feeding trial (d58).

The relative abundances of the top 10 phyla, families, and genera, detected in both the C and T groups at the beginning (d0) and at the end of the feeding trial (d58), are displayed in Figure 1A–C. Overall, Firmicutes, Bacteroidetes, Proteobacteria, Spirochaetes, Tenericutes, Verrucomicrobia, Actinobacteria, and Planctomycetes were the main phyla identified in both the C and T groups at d0 and d58. Concerning temporal changes in microbiota composition, a decrease in the relative abundance of Firmicutes, Proteobacteria, and Planctomycetes and a concomitant increase in Bacteroidetes, Spirochaetes, and Verrucomicrobia were observed in both the C and T groups. Additionally, Fibrobacteres and Cyanobacteria were detected only at day 58 (Figure 1A). Regarding group-specific changes, at the end of the feeding trial (d58), the phyla Tenericutes and Actinobacteria displayed opposite trends in the C and T groups. Specifically, Tenericutes decreased in the C group (from 0.47% at d0 to 0.13% at d58) and increased in the T group (from 0.19% at d0 to 0.31% at d58), whereas Actinobacteria increased in the C group (from 0.47% at d0 to 1.81% at d58) and decreased in the T group (from 2.04% at d0 to 1.29% at d58) (Figure 1A).

At the family level, Ruminococcaceae, Lachnospiraceae, Bacteroidaceae, Spirochaetaceae, Rikenellaceae, Verrucomicrobiaceae, Streptococcaceae, Veillonellaceae, Paraprevotellaceae, and Prevotellaceae represented the top 10 identified families, accounting for more than 70% of the total microbiota (Figure 1B). Among these, Ruminococcaceae, Lachnospiraceae, and Bacteroidaceae showed the highest relative abundances in both the C and T groups over time. In detail, Ruminococcaceae shifted from 17.54% to 17.99% in the C group and from 17.58% to 21.20% in the T group. Lachnospiraceae decreased from 17.47% to 14.55% in the C group and from 21.62% to 17.72% in the T group, whereas Bacteroidaceae shifted from 15.59% to 13.34% and from 12.94% to 11.20% in the C and T groups, respectively. The Spirochaetaceae family increased from 2.14% to 10.76% in the C group and from 2.52% to 8.20% in the T group (Figure 1B). Concerning the Veillonellaceae family, a group-specific temporal trend was revealed. In fact, an increase was observed in the C group (from 1.63% at d0 to 2.51% at d58), while a decrease in the T group (from 4.24% at d0 to 2.30% at d58) was detected. Finally, the Prevotellaceae and Streptococcaceae families, detected only at d58 and d0, respectively, showed the highest relative abundances in the C group (Figure 1B).

As displayed in Figure 1C, 5-7N15, *Treponema*, *Ruminococcus*, *Akkermansia*, *Streptococcus*, *Prevotella*, *Oscillospira*, *Bacteroides*, *Coprococcus*, and YRC22, were the top 10 identified genera. Concerning temporal changes, the genera *Treponema*, *Ruminococcus*, *Bacteroides*, and YRC22 increased in relative abundance, whereas *Oscillospira*, *Coprococcus*, and 5-7N15 decreased. *Akkermansia* remained relatively stable over time. Concerning group-specific differences, the genus *Streptococcus*, detected only at d0, and the genus *Prevotella*, detected only at d58, showed a higher relative abundance in the C group than in the T group.

#### 3.1.2. Alpha and Beta Diversity Indices and Differential Abundance

**Figure 2:**
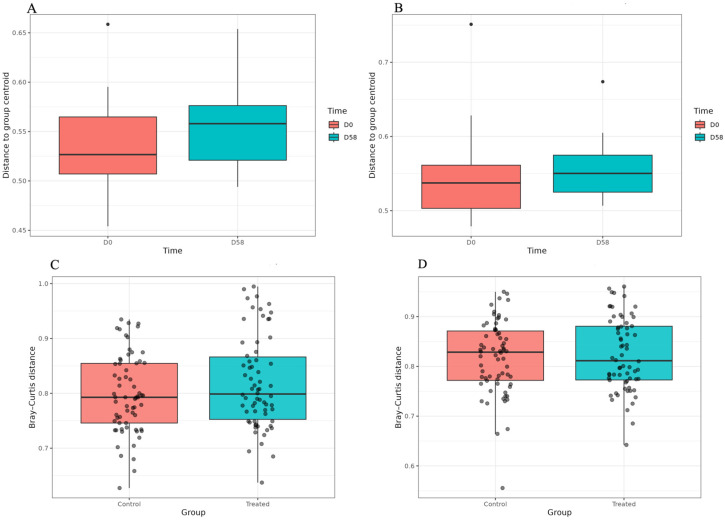
Beta diversity, based on Bray–Curtis distance method, measuring the dissimilarity within the control group (**A**) and treated group (**B**) and between groups at the beginning (d0) (**C**) and at the end (d58) (**D**) of the feeding trial.

The Shannon and 1-Simpson indices within and between groups are plotted in Figure S1A–F. Based on the Wilcoxon test, the α-diversity remained stable over time and between groups. In fact, no statistically significant changes were detected within each group from d0 to d58 (Figure S1A,D) or in between-group comparisons at d0 (Figure S1B,E) and d58 (Figure S1C,F). The results regarding the beta diversity, based on the Bray–Curtis distance method, are depicted in Figure 2, reflecting the dissimilarity within the C group (Figure 2A) and T group (Figure 2B), as well as between groups at d0 (Figure 2C) and d58 (Figure 2D). They revealed different behaviour across time and diets. Additionally, the PCoA results, showing the distribution of samples according to the Bray–Curtis distance, are displayed in Figure S2A–D. Based on the PERMANOVA results, for each group, significant differences were observed when comparing the profiles at d0 and d58 (*p* < 0.05). Additionally, the beta diversity was significantly different between the C and T groups at d58 (*p* < 0.05). Differential abundance at the genus level, within and between groups, was evaluated using MaAsLin2 with total sum scaling (TSS) normalisation and log transformation. Features with a false discovery rate (q-value) ≤ 0.1 were considered significantly associated with time (d58 vs. d0) or diet (C vs. T). In detail, as displayed in Table 1, 15 and 13 genera showed significant temporal changes in the C and T groups, respectively. Among these, although with different magnitudes, a core subset of genera, including *Streptococcus*, *Turicibacter*, *Dorea*, *Prevotella*, *YRC22*, *CF231*, *Fibrobacter*, *Treponema*, and *Succiniclasticum*, showed significant temporal shifts in both the C and T groups. In particular, the genera *Streptococcus*, *Turicibacter*, and *Dorea* decreased at d58, whereas *Prevotella*, *YRC22*, *CF231*, *Fibrobacter*, *Treponema*, and *Succiniclasticum* increased at the same sampling time. In addition to the shared taxa, only in the C group, the genera *Coprococcus* and *Helicobacter* showed a significant decrease from d0 to d58, while *Psychrobacter*, *Bulleidia*, *Sutterella*, and *Bacteroides* increased over time. Conversely, only in the T group, *Oscillospira* and *Anaerovibrio* decreased whereas *Ruminococcus* and *Porphyromonas* increased from d0 to d58. Concerning the comparison between the C and T groups, although none of the genera passed the pre-specified false discovery rate threshold (q < 0.1) for significance, the genera *Acinetobacter*, *Psychrobacter*, and *Bacteroides*, underexpressed in the T group, showed relatively low *p*-values and consistent effect sizes, suggesting a possible trend toward group-specific differences.

### 3.2. Ruminal Microbiota

#### 3.2.1. Taxonomy Classification

**Figure 3:**
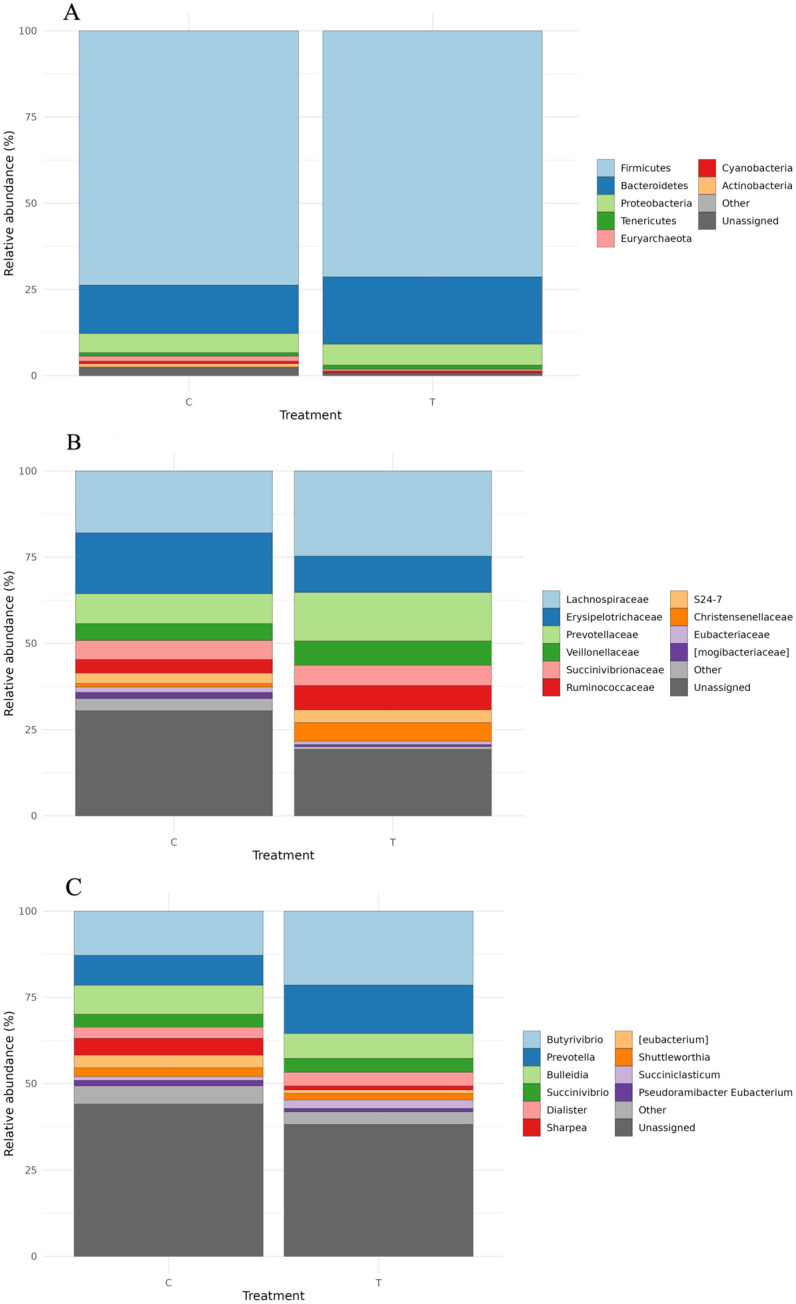
Top 10 phyla (**A**), families (**B**), and genera (**C**) detected in ruminal samples collected from both control (C) and treated (T) groups.

A total of 1.971.469 high-quality sequences—995.675 from C and 975.794 from T samples—were obtained by QIIME2. The sample metadata, denoising statistics, and relative abundances at each taxonomic level (phylum, family, and genus) are reported in Supplementary Data S2. The composition of the ruminal microbiota at the phylum, family, and genus levels, displaying only the top 10 taxa, is reported in Figure 3A–C. Overall, at the phylum level, the microbiota of rumen samples collected from both the C and T groups were dominated by Firmicutes and Bacteroidetes, which together accounted for over 85% of the total sequences. Firmicutes showed a similar relative abundance in C (73.77%) and T (71.39%) samples, whereas the relative abundance of Bacteroidetes was moderately higher in T samples (19.48%) than in C samples (14.00%). Minor phyla included Actinobacteria, Proteobacteria, Cyanobacteria, and Tenericutes (Figure 3A). Based on the family-level taxonomic profiles, as displayed in Figure 3B, the ruminal bacterial communities were dominated by members of the Lachnospiraceae, Erysipelotrichaceae, and Prevotellaceae families. Nevertheless, differences in their relative abundance were observed among groups. In particular, the Lachnospiraceae and Prevotellaceae families were more abundant in T samples, whereas the Erysipelotrichaceae family showed a higher relative abundance in C samples. Veillonellaceae, Succinivibrionaceae, Ruminococcaceae, S24-7, Christensenellaceae, Eubacteriaceae, and Mogibacteriaceae were among the top 10 families. Among these, Succinivibrionaceae and S24-7 showed comparable relative abundances in C and T samples. The Veillonellaceae, Ruminococcaceae, and Christensenellaceae families were more abundant in samples collected from the T group, whereas Eubacteriaceae and Mogibacteriaceae showed higher relative abundances in C samples than in T ones (Figure 3B). At the genus level, *Butyrivibrio*, *Prevotella*, *Bulleidia*, *Succinivibrio*, *Dialister*, *Sharpea*, *Eubacterium*, *Shuttleworthia*, *Succiniclasticum*, and *Pseudoramibacter*_*Eubacterium* were identified as the top 10 taxa. The ruminal bacterial community was dominated by *Butyrivibrio*, which accounted for 12.83% and 21.42% of the sequences in the C and T groups, respectively. *Prevotella*, with a relative abundance of 8.66% in C and 14.06% in T samples, represented the second most abundant genus. *Sharpea* and *Eubacterium* showed higher relative abundances in group C (4.83% and 3.73%, respectively) compared with T (1.12% and 1.05%, respectively). The remaining genera exhibited comparable relative abundances across groups (Figure 3C).

#### 3.2.2. Alpha and Beta Diversity Indices and Differential Abundance

**Figure 4:**
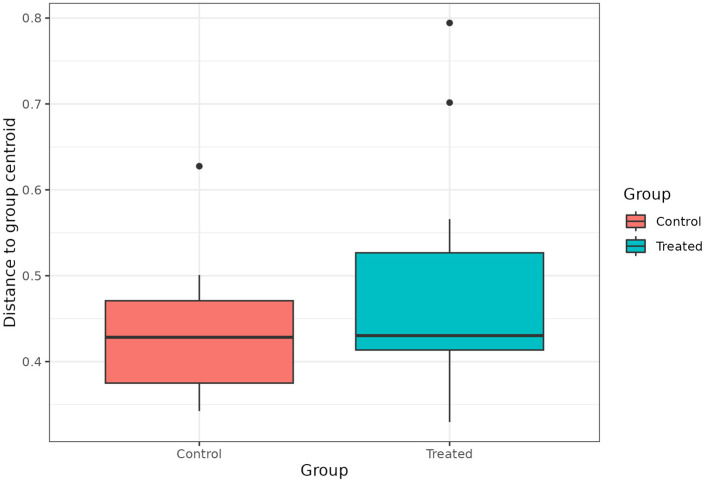
Beta diversity, based on Bray–Curtis distance method, between control and treated rumen samples.

Based on the Wilcoxon test, no statistically significant differences in α-diversity were observed between C and T rumen samples (Figure S3A,B). The beta diversity (Figure 4), based on the Bray–Curtis distance method, showed that the microbiota of the analysed samples diverged in relation to the dietary treatment. The PCoA plot based on the Bray–Curtis distance is reported in Figure S4. Based on the PERMANOVA results, the diet significantly affected the ruminal microbial communities (*p* < 0.05). The differential abundance analysis, performed with MaAsLin2, identified the genera *Eubacterium*, *Sharpea*, *Clostridium*, and *Olsenella* as significantly less abundant in rumen samples collected from the T group (Table 2).

## 4. Discussion

The dynamics and composition of the microbiota associated with the gastrointestinal tract (GIT) in ruminants are affected by several factors, such as host age, diet, genetics, and interactions with the environment [29,30,31,32]. Among these, diet has the greatest influence on the GIT microbiome, with the potential to alter both the microbial diversity and the abundance of individual taxa [29,31,33,34]. Recently, novel feeding strategies based on the integration of agro-industrial by-products have been explored in modern livestock systems, with the aim of supporting animal welfare and promoting sustainability through circular economy principles [35]. Although mixtures of hulls, twigs, leaves, and fragments of the shells and kernels of pistachio have been investigated as dietary supplements for ruminants, to the best of our knowledge, few studies have evaluated pistachio skin as a feeding ingredient [18,19]. In a previous study, Musati and co-workers [21] demonstrated the high content of bioactive compounds, like polyphenols and tannins, in a feeding regime supplemented with pistachio skin. In particular, the diet enriched with 12% of pistachio skin showed polyphenol and tannin content that was approximately three-fold and five-fold higher, respectively, than in the control diet. Based on the well-known antimicrobial activity of these compounds, the present exploratory study, by applying a metagenomics approach, aimed to deepen our knowledge of the effects of the inclusion of pistachio skin on both the faecal and ruminal microbiota of healthy lambs. Data revealed that the experimental diet did not change the overall bacterial diversity. In fact, for both the faecal and ruminal microbial communities, no significant differences in alpha diversity were observed. However, according to both the beta diversity analysis and MaAsLin2 results, only specific taxa, rather than the entire microbial community, were affected during the feeding trial. This is consistent with other studies that showed that, while the overall diversity remained stable, certain bacteria were influenced by the supplementation of agro-industrial by-products, especially those rich in antimicrobial compounds such as polyphenols [6,9,13,22]. Regarding the faecal microbiota, the observed changes appeared to be primarily driven by temporal dynamics rather than by pistachio skin supplementation. Only a potential trend toward group-specific differences was associated with the genera *Acinetobacter*, *Psychrobacter*, and *Bacteroides*, based on the relative abundance.

Considering the ruminal microbiota composition, specific taxa were differentially represented between groups, suggesting a possible interaction of polyphenols, tannins, and other plant secondary metabolites in pistachio skin with rumen microorganisms [36,37]. In small ruminants, the use of by-products rich in polyphenols, along with influencing rumen fermentation parameters, can induce a reduction in the abundance of microbial groups associated with fibre degradation and protein fermentation [6]. In the present study, the genera *Eubacterium*, *Sharpea*, *Clostridium*, and *Olsenella* were underexpressed in the rumen microbiota of lambs fed the diet enriched with 12% of pistachio skin. Among these, the genus *Eubacterium* is recognised for the ability to degrade complex plant cell wall components and to produce short-chain fatty acids (SCFAs), particularly butyrate and acetate, through the fermentation of cellulose, hemicellulose, and related polysaccharides [38,39]. Although *Eubacterium* was significantly lower in the T group than in the C one, no significant alterations in the SCFA profile were observed by Musati and co-workers [21] in the same rumen samples. *Olsenella* is involved in carbohydrate fermentation pathways, particularly the fermentation of easily fermentable sugars into organic acids, including lactic, acetic, and formic acids [40,41]. The reduced relative abundance of *Olsenella* in the T group was not supported by the SCFA profiles reported by Musati et al. [21]. In fact, the authors did not reveal significant differences between control and treated rumen samples [21]. Nevertheless, the underexpression of the genera *Eubacterium* and *Olsenella* revealed in the present study is in line with previously reported data focused on feeds containing phenolic-rich by-products. Similarly, Daghio and co-workers [42], by characterising and comparing the microbial communities of rumen digesta from lambs fed a diet supplied with hazelnut skin or a control diet, showed that both *Olsenella*- and *Eubacterium*-related groups displayed lower relative abundances in samples from lambs fed with the experimental diet compared to the control one. The *Sharpea* genus, which includes lactate-producing species, was recently associated with hydrogen production in the rumen. In particular, as reported by Kittelmann and co-workers [43], *Sharpea*, mainly *Sharpea azabuensis*, is associated with low methane emissions in sheep. Similarly, using deep sequencing metagenome and metatranscriptome datasets in combination with 16S rRNA gene amplicon sequencing, the enrichment of lactate-producing *Sharpea* spp. was revealed in low-methane-yield sheep compared to their high-methane-yield counterparts [44]. These bacteria are associated with shifts in volatile fatty acid production, such as propionate, which represent a more efficient energy substrate for the host and can reduce hydrogen availability for methanogens, thereby lowering methane production. By combining our microbial profiling with the ruminal fermentation parameters reported by Musati and co-workers [21], it can be concluded that the lower relative abundance of *Sharpea* in the pistachio-supplemented group in the present study was not associated with a significant change in gas and methane production, indicating that the pistachio skin did not influence ruminal fermentation parameters [20,21].

Regarding the main limitations of the present study, the relatively small number of animals per group (*n* = 12), albeit common in exploratory microbiome analyses, may limit the generalisability of the results, especially considering the inter-individual variability of ruminal and faecal microbiota. Additionally, the analysis of single rumen samples, collected at slaughter, provides only a single-timepoint snapshot, limiting the assessment of temporal dynamics and potential diet-induced changes over time. Another limitation of the present study is the use of relative abundance data from 16S rRNA amplicon sequencing, which may introduce compositional biases and does not allow the quantification of absolute changes in community structure.

## 5. Conclusions

The inclusion of pistachio skin in the lamb diet did not significantly affect the faecal microbiota. The observed changes were mainly driven by temporal dynamics rather than by the diet. However, in the ruminal microbiota, specific bacterial taxa were differentially represented, indicating a possible diet-related effect. Although the present study supports the potential use of pistachio skin as a sustainable feed ingredient in ruminant diets, further studies are needed to explore lambs’ gastrointestinal functionality.

## Figures and Tables

**Table 1:** Significant results from the MaAsLin2 differential abundance analysis within and between C and T faecal samples.

Taxon	Metadata	Value	Coefficient	*p*-Value	q-Value
*Prevotella*	C	d58	2.8234	<0.001	<0.001
*Streptococcus*	C	d58	−3.7759	<0.001	<0.001
*YRC22*	C	d58	1.7078	<0.001	<0.001
*CF231*	C	d58	2.0935	<0.001	0.002
*Treponema*	C	d58	2.7294	<0.001	0.003
*Turicibacter*	C	d58	−3.1030	<0.001	0.004
*Sutterella*	C	d58	1.4836	0.001	0.007
*Coprococcus*	C	d58	−1.6222	0.002	0.008
*Fibrobacter*	C	d58	2.2336	0.003	0.012
*Psychrobacter*	C	d58	1.1879	0.008	0.029
*Bacteroides*	C	d58	1.7095	0.014	0.046
*Dorea*	C	d58	−1.0511	0.018	0.053
*Bulleidia*	C	d58	1.1905	0.023	0.062
*Helicobacter*	C	d58	−1.5703	0.027	0.067
*Succiniclasticum*	C	d58	0.6399	0.041	0.095
*YRC22*	T	d58	2.4850	<0.001	0.005
*Succiniclasticum*	T	d58	1.6040	<0.001	0.007
*Prevotella*	T	d58	3.0522	<0.001	0.007
*Porphyromonas*	T	d58	1.6596	0.001	0.011
*CF231*	T	d58	0.8825	0.002	0.012
*Streptococcus*	T	d58	−2.8790	0.003	0.015
*Turicibacter*	T	d58	−2.0478	0.003	0.015
*Oscillospira*	T	d58	−1.9932	0.003	0.015
*Treponema*	T	d58	1.8721	0.008	0.031
*Anaerovibrio*	T	d58	−1.5924	0.032	0.093
*Dorea*	T	d58	−1.1835	0.017	0.061
*Fibrobacter*	T	d58	1.1740	0.019	0.062
*Ruminococcus*	T	d58	1.0149	0.035	0.094
*Acinetobacter*	Group	T	−1.7881	0.013	0.216
*Psychrobacter*	Group	T	−0.8359	0.007	0.216
*Bacteroides*	Group	T	−1.4230	0.023	0.243

**Table 2:** Significant results from the MaAsLin2 differential abundance analysis between C and T rumen samples.

Taxon	Metadata	Value	Coefficient	*p*-Value	q-Value
*Eubacterium*	Group	T	−1.7528	<0.001	0.007
*Sharpea*	Group	T	−2.3672	0.002	0.019
*Clostridium*	Group	T	−1.5884	0.003	0.025
*Olsenella*	Group	T	−1.47182	0.005	0.026

## Data Availability

The raw 16S rRNA gene sequencing data are openly available in the NCBI BioProject database under accession numbers PRJNA1115961 and PRJNA1368722.

## Supplementary Materials

The following supporting information can be downloaded at https://www.mdpi.com/article/10.3390/microorganisms14020358/s1. Figure S1. α-diversity of faecal samples based on Shannon and 1-Simpson indices within (A and D) and between groups at d0 (B and E) and d58 (C and F) sampling times. Figure S2. Principal coordinate analysis (PCoA) based on Bray–Curtis distances, showing the distribution of samples within the control (A) and treated (B) groups and between groups at the beginning (d0) (C) and at the end (d58) (D) of the feeding trial. Figure S3. α-diversity based on Shannon (A) and 1-Simpson (B) indices between rumen samples collected from control and treated groups. Figure S4. Principal coordinate analysis (PCoA) based on Bray–Curtis distances, showing the distribution of rumen samples collected from control and treated groups. Supplementary Data S1: Faecal samples metadata, denoising statistics, and relative abundances at each taxonomic level (phylum, family, and genus). Supplementary Data S2: Rumen samples metadata, denoising statistics, and relative abundances at each taxonomic level (phylum, family, and genus).
