# Supplementary material for: Effect of By-Products from Pistachio Skin on Gastrointestinal Microbiota of Healthy Lambs as Sustainable Feeding Ingredient
Source: Microorganisms. 2026 Feb 3;14(2):358. doi: 10.3390/microorganisms14020358 (PMC12942887; doi:10.3390/microorganisms14020358)
Supplement: Supplementary file 1 [file microorganisms-14-00358-s001.zip › Supplementary materials.pdf]

Supplementary Material

Effect of by-products from pistachio skin on gastrointestinal microbiota of healthy lambs as sustainable feeding ingredient

Georgiana Bosco[1], Amanda Vaccalluzzo[2], Nunziatina Russo[1,2], Alessandra Pino[1,2*], Cinzia Caggia[1,2] and Cinzia Lucia Randazzo[1,2]

[1] Department of Agriculture, Food and Environment, University of Catania, 95123 Catania, Italy; georgiana.bosco@phd.unict.it (G.B.); nunziatinarusso83@gmail.com (N.R.); cinzia.caggia@unict.it (C.C.); cinzia.randazzo@unict.it (C.L.R.)

[2] ProBioEtna srl, Spin off of the University of Catania, 95123 Catania, Italy; amanda.vaccalluzzo@unict.it

* Correspondence: alessandra.pino@unict.it

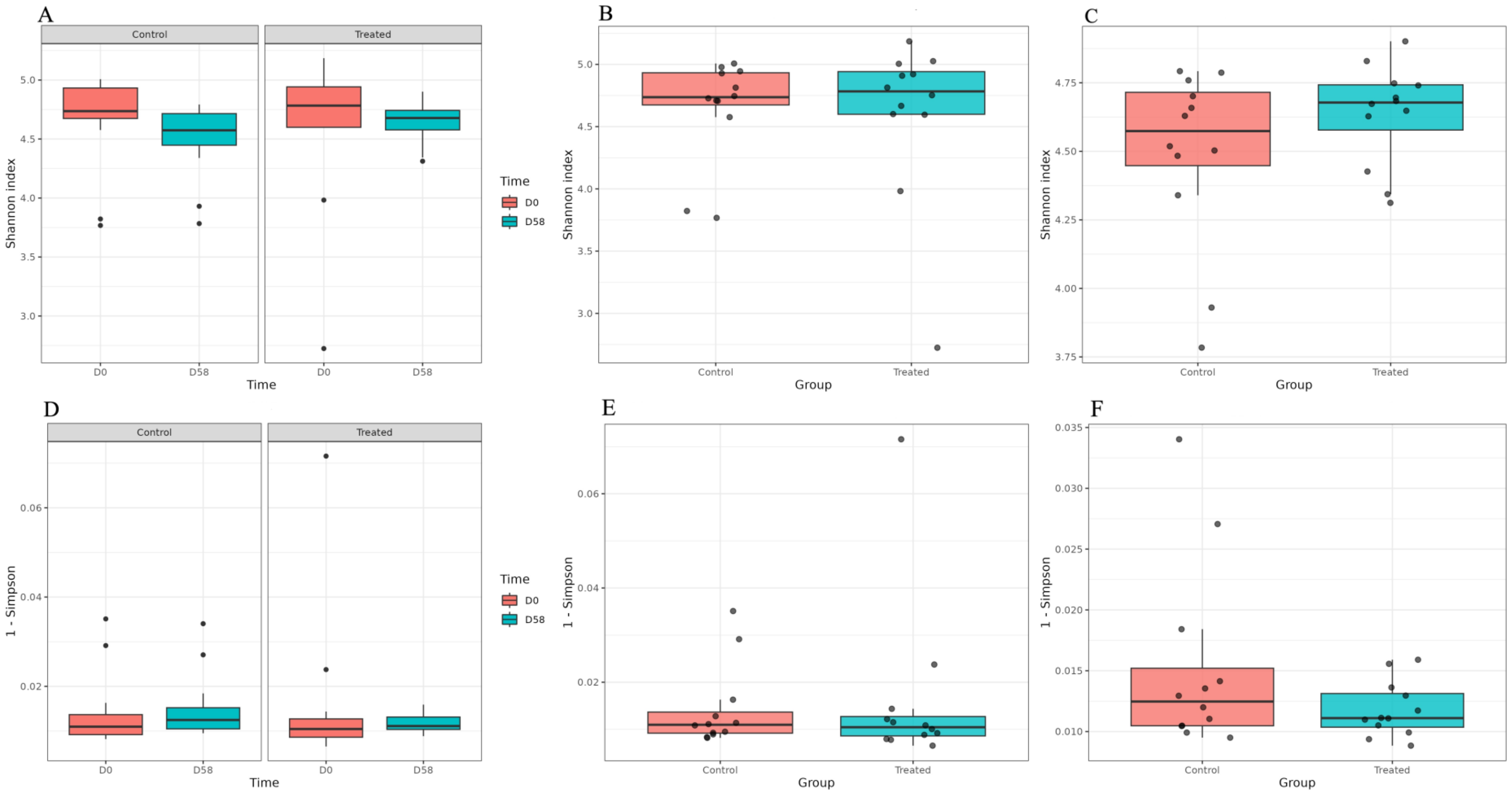

Figure S1. α-diversity, of faecal samples, based on Shannon and 1-Simpson indices within (panels A and D) and between groups at d0 (panels B and E) and d58 (panels C and F) sampling times.

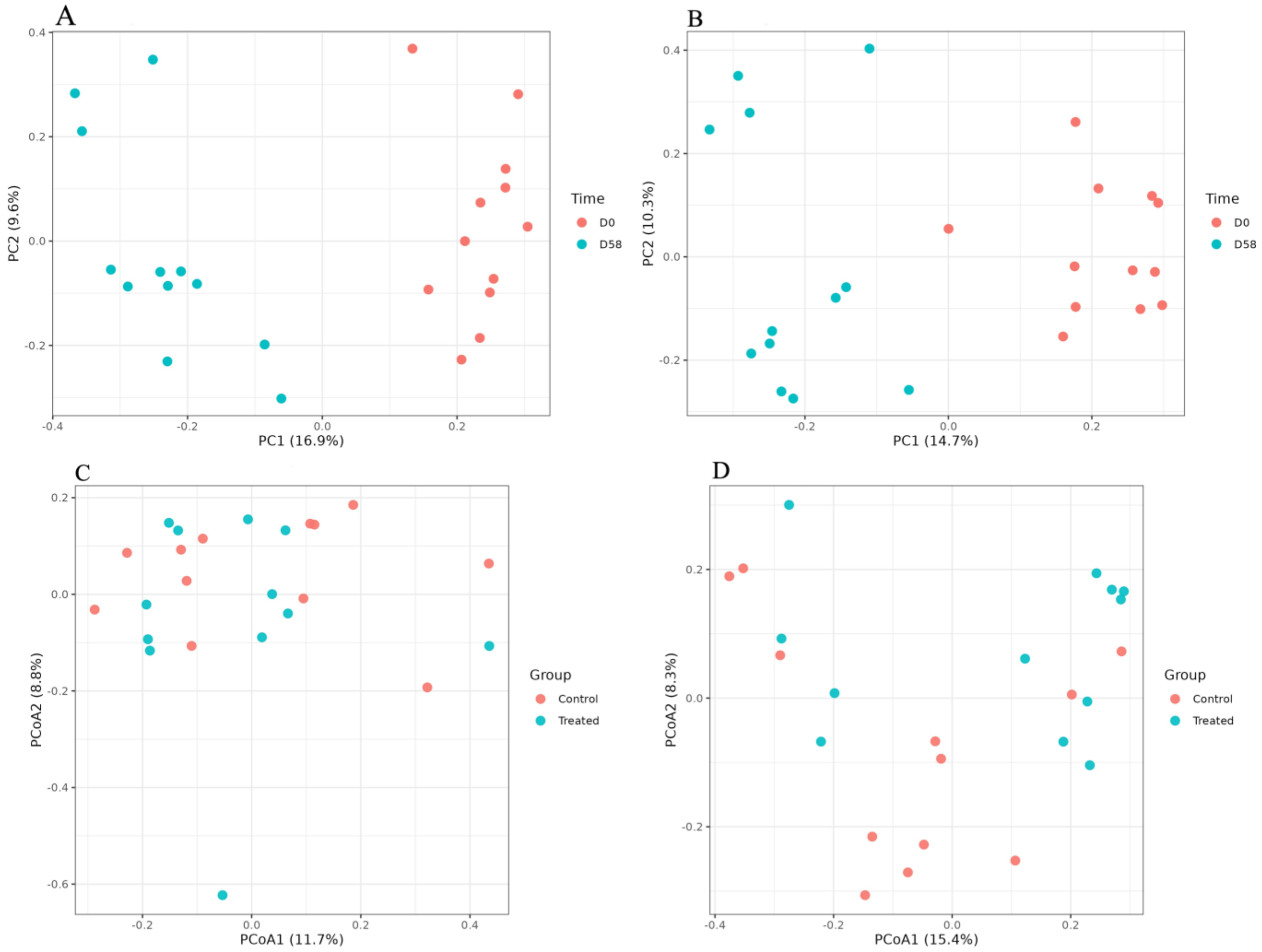

Figure S2. Principal Coordinate Analysis (PCoA) based on Bray–Curtis distances showing the distribution of samples within control (panel A) and treated (panel B) groups and between groups at the beginning (d0) (panel C) and at the end (d58) (panel D) of the feeding trial.

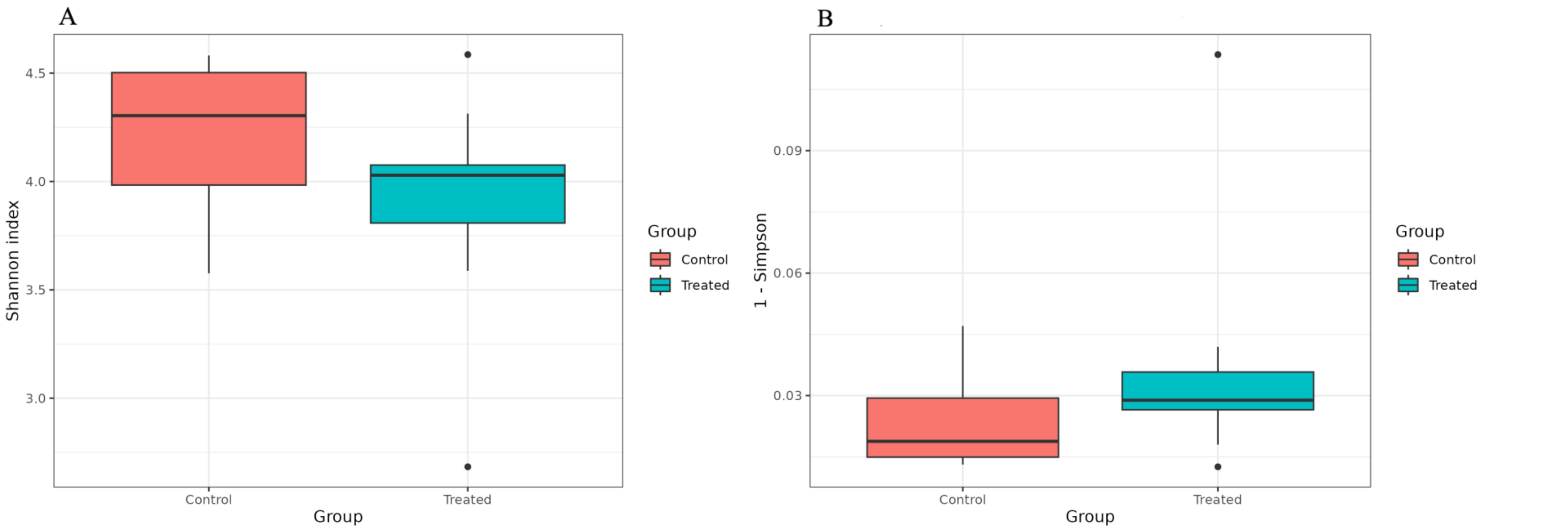

Figure S3. α-diversity based on Shannon (panel A) and 1-Simpson (panel B) indices between rumen samples collected from Control and Treated groups.

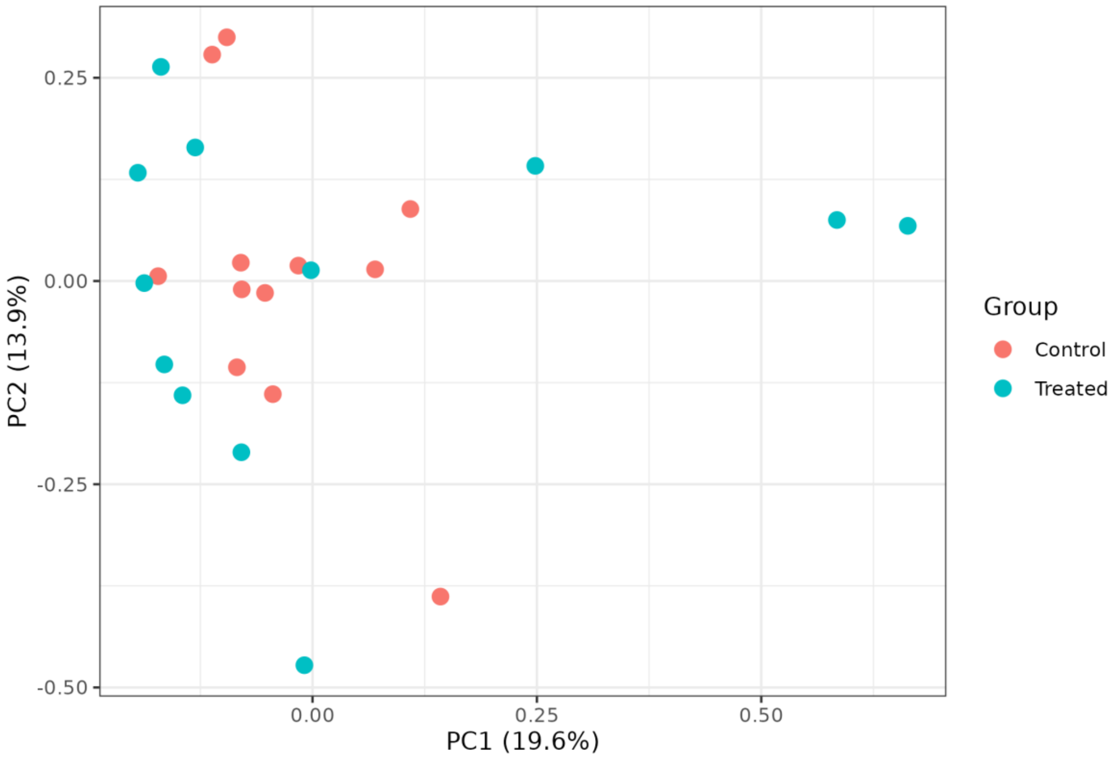

Figure S4. Principal Coordinate Analysis (PCoA) based on Bray–Curtis distances showing the distribution of rumen samples collected from Control and Treated groups.
